# Global posture reeducation compared with segmental muscle stretching exercises in the treatment of fibromyalgia: a randomized controlled trial

**DOI:** 10.1186/s13063-023-07422-w

**Published:** 2023-06-07

**Authors:** Luciana Akemi Matsutani, Adriana de Sousa do Espírito Santo, Marina Ciscato, Susan Lee King Yuan, Amelia Pasqual Marques

**Affiliations:** Departamento de Fisioterapia, Fonoaudiologia e Terapia Ocupacional, Faculdade de Medicina, Universidade de Sao Paulo, Rua Cipotânea 51, Cidade Universitária, São Paulo, SP Brazil

**Keywords:** Fibromyalgia, Physical therapy modalities, Muscle stretching exercises, Cognitive behavioral therapy

## Abstract

**Background:**

Muscle stretching exercises preserve corporal flexibility and decrease the retraction and shortening of myofascial and articular structures. These exercises are recommended for the treatment of fibromyalgia (FM). The purpose of the study was to verify and compare the effect of muscle stretching exercises on FM patients based on the global posture reeducation method against segmental muscle stretching exercises, both used in concert with an educational approach rooted in cognitive behavioral therapy.

**Methods:**

Forty adults with FM were randomly allocated into two groups: global and segmental. The two kinds of therapies were performed in 10 individual sessions once a week. Two assessments were made: one at baseline and one at the end of therapy. The primary outcome variable was pain intensity (Visual Analog Scale). The secondary outcome variables were multidimensional pain (McGill Pain Questionnaire), the pain threshold at tender points (dolorimetry), attitudes toward chronic pain (Survey of Pain Attitudes-Brief Version), body posture (Postural Assessment Software Protocol), postural control (Modified Clinical Test of Sensory Interaction on Balance), flexibility (sit-and-reach test), the impact of FM on quality of life (Fibromyalgia Impact Questionnaire, FIQ), and self-reported perceptions and body self-care.

**Results:**

At the end of treatment, there were no statistically significant differences between the groups in the outcome variables. Furthermore, the groups presented lower pain intensity (baseline vs. final; global group: 6 ± 1.8 vs. 2.2 ± 1.6 cm, *p*<0.01; segmental group: 6.3 ± 2.1 vs. 2.5 ± 1.7 cm, *p*<0.01), higher pain threshold (*p* ≤ 0.01), lower total FIQ score (*p* < 0.01), and greater postural control (*p* < 0.01) after treatment.

**Conclusions:**

Muscle stretching exercises based on global posture reeducation and segmental muscle stretching exercises, both used in concert with an educational approach rooted in cognitive behavioral therapy, reduced the pain intensity and impact of FM on quality of life. These exercises also improved FM patients’ pain threshold at tender points, attitudes toward chronic pain, and postural control. There were no differences between global posture reeducation and segmental muscle stretching exercises.

**Trial registration:**

ClinicalTrials.gov NCT02384603. Registered on 10 March 2015.

## Background

Fibromyalgia (FM) is a syndrome characterized by widespread chronic pain. It has been linked to a multitude of symptoms, primarily nonrestorative sleep, fatigue, and depression [1, 2]. The symptoms typically affect women (prevalence rates of 3.7% and 0.9% in the female and male populations, respectively) [3].

Among people with FM, there seems to be an amplification of pain and other sensory stimuli due to neuroplastic changes in the central nervous system [4], as well as neuroendocrine changes in the hypothalamic–pituitary–adrenal axis and the autonomic nervous system [5] related to difficulties with stress management [6].

Physiotherapy or physical activity/exercise and multicomponent therapy (at least one educational or other psychological therapy with at least one exercise therapy) are recommended for the treatment of FM [7]. In particular, muscle stretching exercises decrease retraction and the shortening of myofascial and articular structures and preserve corporal flexibility, which is one component of physical fitness [8]. These exercises are recommended for the treatment of FM. However, there is little scientific evidence on the treatment of FM compared to aerobic exercise [9], and muscle stretching exercise trials are needed [10]. One method of muscle stretching exercises based on body posture, used extensively in other populations [11], addresses muscular chains through a technique known as global posture reeducation, originally developed by Philippe-Emmanuel Souchard [12]. Marques et al. [13] conducted a descriptive study of 20 FM patients, in which they evaluated the effect of muscle stretching exercises based on global postural reeducation. They found that 13 patients (65%) reported very good and good improvement, 5 patients (25%) reported regular improvement, and 2 patients (10%) reported no improvement at the end of six sessions. It is relevant to use this technique in FM, as treatments improving postural alignment have been shown to play a role in the prevention and treatment of pain in musculoskeletal disorders [14, 15].

The reason muscle stretching exercises might work for individuals with FM is based on the assumption that pain is a stressor [16, 17] that can generate an emotional state associated with depression (sadness, anger, guilt, or fear), which impairs quality of life [18, 19]. According to Miranda et al. [20], when there is difficulty in managing stress, “the person contracts, compacts, tightens, shrinks or holds.” Adults with FM show a greater increase in muscular activity under experimentally imposed mental stress than controls [21]. This significant increase in muscle activity could lead to the activation of parallel mechanisms of pain induction, such as myofascial retraction [20, 21]. Muscle stretching exercises can help with relaxation and, therefore, decrease pain [8].

Individuals with FM must actively cooperate with health professionals to achieve increased autonomy, which in turn, contributes to an improved quality of life. Learning about FM, healthy behaviors, and positive perspectives is also important for individuals with FM, as these factors could increase resilience, reduce suffering, and improve quality of life [22].

Patient education combined with exercise is recommended for the treatment of FM [7, 23]. One method of learning, known as cognitive behavioral therapy, consists of two elements: (a) behavioral, which is based on the theories of classical and operant conditioning and is focused on aspects of the environment that modulate pain and other symptoms, and (b) cognitive, which is based on Beck’s cognitive model of depression, in which the way individuals structure the world influences their pain, other symptoms, functionality, and emotions. Cognitive behavioral therapy is an active approach that focuses on understanding, training, and applying health skills in real-life situations [24, 25], along with changing negative thoughts about pain and function [26].

The objective of this study was to verify and compare the effect of muscle stretching exercises on FM patients based on the global posture reeducation method against segmental muscle stretching exercises, both used in concert with an educational approach rooted in cognitive behavioral therapy.

## Methods

This is a randomized parallel trial and a qualitative study developed by the Laboratório de Investigação Fisioterapêutica Clínica, Departamento de Fisioterapia, Fonoaudiologia e Terapia Ocupacional, Faculdade de Medicina, Universidade de Sao Paulo. The study and informed consent terms were both approved by the Research Ethics Committee of the Faculdade de Medicina, Universidade de Sao Paulo, and both were registered at *ClinicalTrials.gov* (NCT02384603)—start date of recruitment: February 2016. This work was completed in March 2018.

Individuals with FM who were residents in the metropolitan area of São Paulo, Brazil, and referred to the Departamento de Fisioterapia, Fonoaudiologia e Terapia Ocupacional, Faculdade de Medicina, Universidade de Sao Paulo, were enrolled in the study. The inclusion criteria were adults between 30 and 60 years of age, a diagnosis of FM according to the American College of Rheumatology (ACR) 2010 criteria [27], and the presence of at least 11 of 18 tender points. The exclusion criteria were simultaneous physical therapy, the presence of joint deformities, and an inability to walk independently. A total of 101 eligible participants were identified.

The sample size calculation (SigmaPlot 12.3 software, Systat Software Inc.) was performed based on the visual analog pain scale data included in a study conducted by the research group [28]: paired *t*-test, a detectable change of 2.0 ± 2.9 cm between baseline and posttreatment, and a level of significance of 0.05 and power of 0.80, resulting in a sample size of 19 adults in each group. The study hypothesis was that both methods of muscle stretching exercises, used in concert with an educational approach, could be beneficial to FM patients.

To balance the number of participants in the two groups, block randomization (subjects in blocks of four at a time) was used for the sequence generation process [29]. An excerpt from a table of random two-digit numbers was created using SigmaPlot 12.3 software (Systat Software Inc.). Sequentially numbered, opaque, sealed envelopes were used to conceal the group allocations prior to the assignment. The researcher in charge of randomly assigning the participants was not blinded and did not participate in the outcome assessment evaluation, but participated in the intervention or analysis. The outcome evaluator was blinded to the randomization process and the participants’ allocation. The evaluator was a physiotherapist who received training in this kind of evaluation. The participants were informed about their designated group and were instructed not to inform the evaluator. The participants also did not contact each other and were blinded to the hypothesis of the study. It was not possible to hide each participant’s designation from the physiotherapist involved in the treatment.

### Assessment

The evaluation was made at the beginning and end of the 10-week treatment period. The modified diagnostic ACR 2011 criteria are indicated as an evaluation instrument in clinical and epidemiological studies [30]. The criteria, which were applied during an interview, consisted of the following: (I) the Widespread Pain Index (WPI), representing the sum of body sites referred to as painful in the last week and ranged from zero to 19, and (II) the Symptom Severity Score (SSS), representing the sum of the scores of other disorders (fatigue, cognitive problems, sleep disorders in the last week, and three somatic symptoms, i.e., abdominal pain, depression, and headache in the last 6 months) and can vary from zero to 12. A patient satisfies the ACR 2011 criteria for FM with the following scores: WPI ≥ 7 and SSS ≥ 5 or WPI of 3–6 and SSS ≥ 9.

#### Primary outcome variable

##### Pain intensity

Pain intensity was evaluated with the Visual Analog Scale for Pain (VAS) [31, 32] consisting of a 10-cm long horizontal line, with “no pain” on the far left and “unbearable pain” on the far right. Each patient was asked to mark the line indicating the pain intensity at the moment. The minimum score was zero, and the maximum was 10.

#### Secondary outcome variables

##### Multidimensionality of pain

The pain dimensions were assessed with the McGill Pain Questionnaire (MPQ) [33], which consisted of 78 words describing the quality of pain and were organized into 20 groups that formed three dimensions: sensory, affective, and evaluative. Each word was assigned an intensity value on a numerical scale of one to five points. A patient was asked to choose the word in each group that described the pain at the moment, with “none” as an option. The maximum scores of the dimensions were as follows: sensory = 41, affective = 14, and evaluative = 5. The higher the score, the greater the intensity of the pain.

##### Pressure pain threshold at tender points

The pressure pain threshold at tender points was evaluated by dolorimetry with a dolorimeter (FDX, Wagner Instruments®) that measured the pain threshold (i.e., the value of the lowest pressure at which a patient-reported pain). The patients’ pain threshold was assessed at 18 tender points according to the ACR FM classification criteria [1] by applying progressively greater pressure perpendicular to the surface of the skin at a rate of approximately 1 kg/cm^2^/s. Each patient was instructed to state when the feeling of pressure turned into pain. With the patient seated, the tender points of the base of the occipital, lower cervical, trapezius, supraspinatus, second costochondral joint, lateral epicondyle, and medial border of the knee were assessed bilaterally. The patient was asked to stay upright to evaluate the tender points of the gluteus and the greater trochanter.

##### Attitudes of patients toward chronic pain

The Survey of Pain Attitudes (SOPA)-Brief Version [34] consists of 30 items corresponding to seven domains of attitudes, beliefs, and behaviors toward pain: Control, Emotion, Disability, Harm, Medication, Solicitude, and Medical cure. In this study, the evaluator used the instrument, and the patients indicated their agreement with each assertion on a five-point Likert-type scale ranging from zero to four. The score of each domain was calculated as the sum of the points of the answers to each item divided by the number of items answered. The final average score for each domain ranged from zero to 4. There are no cutoff points and no right or wrong answers. The guidelines define certain more desirable responses that are considered hypothetically more adaptive by the author of the inventory. The desirable score orientations for each domain after adjustment were as follows: Control = 4, Emotion = 4, Disability = 0, Harm = 0, Medication = 0, Solicitude = 0, and Medical cure = 0.

##### Impact of FM on quality of life

The Fibromyalgia Impact Questionnaire (FIQ) was used [35] to measure the impact of FM on patient’s quality of life. This scale consisted of 19 items organized into 10 questions about the most recent week, which were then used to evaluate physical function, well-being, lack of work, difficulty with work, and intensity of symptoms. The calculation of the total score was as follows: Question 1 = (sum of the values of the items) × 10 ÷ 30 + Question 2 = (inversion of the value) × 10 ÷ 7 + Question 3 = (value) × 10 ÷ 7 + Questions 4 to 10 = the value of each question. The maximum total score was 100. The higher the score, the worse a patient’s quality of life.

##### Body posture

Body posture evaluation was conducted by photogrammetry with the Postural Assessment Software Protocol (PAS/SAPO) [36, 37]. The camera (Cyber-shot DSC-W230, Sony®) was positioned on a tripod at a distance of 1.50 m with the lens at the height of the patient’s umbilical line. The following body posture measurements were analyzed and interpreted according to the protocol introduced by Duarte et al. [38]: right lateral view: (a) horizontal alignment of the head (position of head in relation to trunk, wherein lower values refer to greater head forward position); (b) vertical alignment of trunk (positive values indicate anterior trunk tilt, while negative values indicate the opposite); (c) vertical body alignment (positive values indicate anterior body tilt, while negative values indicate the opposite); anterior view: (d) horizontal alignment of head; (e) horizontal alignment of acromia; and (f) horizontal alignment of the anterior superior iliac spine. For the last three measurements, the software reference value is zero, and the positive and negative values indicate tilting to the right and left, respectively.

##### Flexibility

Flexibility was assessed with the sit-and-reach test [39, 40], which utilized a Wells bench (Sanny®). Each patient was asked to sit on the floor with legs extended, without shoes, feet apart, leaning against the bench, arms at shoulder level, and hands overlapping. The patient reached as far as possible along the measurement line of the Wells bench, keeping the knees in extension. The measure of the second attempt was recorded. The higher the value, the greater the flexibility of the patient.

##### Postural control

The Modified Clinical Test of Sensory Interaction on Balance (mCTSIB) was used to assess postural control and was performed on a pressure platform (NeuroCom Balance Master®) [41]. This test evaluated static balance under four sensory conditions while each patient was instructed to stand quietly erect, with arms straight alongside the body and with bare feet in the position recommended by Neurocom: eyes open and stable surface, eyes closed and stable surface, eyes open and unstable surface, eyes closed and unstable surface. The center of gravity sway velocity (degrees/second) corresponds to the sum of the anteroposterior and mediolateral sway measured for 10 seconds per trial. The mean value of the three measures of the center of gravity sway velocity under each of the four sensory conditions was considered. The sum of the means divided by the number of conditions (mCTSIB mean) was used. The mean value was provided by version 8.3.0 of the Balance Master® operating system. In this measurement tool, the higher the center of gravity sway velocity, the worse the postural control.

##### Perception and body self-care

*Audio recording of the story*: All the patients reported on how they perceived their bodies and posture and took care of their bodies. The reports were audio-recorded for transcription.

#### Treatments

The FM patients attended 10 individual treatment sessions, each approximately 80 min long, once a week for 10 weeks. To maximize adherence to treatment, individualized attention, such as phone calls after missed sessions, was given to each patient. More than three consecutive absences were considered discontinuation of therapy.

A physiotherapist with 15 years of experience in FM clinical research and in the educational area, who is a specialist in the global posture reeducation method developed by Philippe-Emmanuel Souchard [11], led the cognitive behavioral therapy-based educational approach and muscle stretching exercises for both groups. She was trained in this approach by the researcher-in-chief (PhD in Experimental Psychology) and had a background in teaching coping skills regarding health for adults and elderly adults.

The treatments are described in Table 1. In all sessions, the cognitive behavioral therapy-based educational approach was employed. This approach involved a structured educational methodology [42] based on the following three references: (I) the conditions offered by the physiotherapist, (II) the expected outcome of the activity, and (III) the real outcome of the activity of the week. The acquisition and maintenance of coping skills were enhanced by the convergence of items II and III. In addition, the physiotherapist worked with patients to identify dysfunctional and negative thought patterns, as well as the underlying maladaptive attitudes or beliefs fueling those thoughts. Positive perspectives were emphasized with the patients.

**Table 1:** Report on muscle stretching exercises used in concert with an educational approach

Each session was divided into two parts: (I) Educational/cognitive behavioral therapy (about 30–40 min) - Conditions offered by the physiotherapist: Talk about FM and difficulties in daily activities because of FM (positive perspectives). - Expected product of the activity: Adoption of body self-care resources to reduce pain and other symptoms (incorporation into daily life). - Actual product of the activity of the week: Verified in conversation with the patient. (II) Exercises (about 30–40 min), except for the first session. *Session 1* • Conditions offered by the physiotherapist: Talk about FM and difficulties in daily activities because of FM. Delivery of the FM guidance booklet based on Marques et al. [43]. • Activities performed: Adding body self-care to the daily activities. *Session 2* • Conditions offered by the physiotherapist: Perceptions of the body parts and movements: proprioception (body and movement) and adaptation to the difficulties presented by the patient. • Activities performed - Contact of the feet with the ground; recognition of foot pressure on the ground; differences and similarities in relation to both feet - Perceptions of the positioning of shoulders, trunk, and head with orientation for adopting good body posture - Foot sensitization exercises: massage with a tennis ball and a piece of bamboo, with the feet on the ground - Exercises in body weight transfer in the frontal and sagittal planes, as well as perceiving the body weight distributed in a similar way between the feet - These exercises were done with the eyes open, eyes closed, and in front of a mirror. *Session 3* • Conditions offered by the physiotherapist: Guidance for body perception (proprioception) on a stretcher. • Activities performed: Perceptions of the body and its supports on the stretcher. In dorsal decubitus, the patients were instructed to keep their eyes closed, relax any tense muscles, and pay attention to their breathing (achieving a slow and constant rhythm between inhalation and exhalation). The physiotherapist helped guide their perceptions, calmly describing each body segment from toe to head.	
*Sessions 4–10*	
**GLOBAL GROUP** • Conditions offered by the physiotherapist: Preparatory maneuvers of manual therapy associated with breathing for the stretching exercises in muscle chains. In dorsal decubitus position with a relaxed body. Myofascial release of the shoulder and pelvic girdles, anterior chest, and paraspinal muscles associated with breathing. • Activities performed: During the session, care was taken to avoid postural compensation (due to increased tension in response to muscle shortening) in specific body segments and to restrict exercise to a minimum of discomfort. - First position of the global posture reeducation method, 15 min long: to stretch the posterior muscle chain, the participant remained in the dorsal decubitus position. The goal was to reach the final stretch position with the arms “adducted” and the lower limbs with a hip flexion to 90° supported in a specific band of the global posture reeducation stretcher. The knee extension was progressively performed (respecting the participant’s limit) with the ankle in dorsiflexion, keeping the occipital, lumbar region, and sacrum stabilized. - Second position of the global posture reeducation method, 15 min long: The anterior muscle chain was stretched with the participant in dorsal decubitus, arms abducted to about 30°, and the forearms in a supine position. The pelvis and lumbar segment remained stabilized. The hips were flexed, abducted, and rotated laterally with the plantar regions of the feet in contact. The lower limbs were progressively “extended” to the maximum extension of the knees, keeping the tibiotarsus at an angle of 90°; at the end of the posture, the arms reached approximately 140° of abduction.	**SEGMENTAL GROUP** • Conditions offered by the physiotherapist: Preparatory maneuvers of manual therapy associated with breathing for the stretching exercises. In dorsal decubitus position with a relaxed body. Myofascial release of the shoulder and pelvic girdles, anterior chest, and paraspinal muscles associated with breathing. • Activities performed: Three muscle stretching exercises, repeated three times each, gradually improved in the course of sessions according to participant tolerance, no more than five times each. The participants performed static segmental muscle stretching exercises with the therapist’s assistance. The exercise intensity was gently increased gradually to the point of minimum discomfort, and each position was held for 30 s, with the same time for rest between repetitions. *Session 4* • Activities performed - Paravertebral: In dorsal decubitus, the patients flexed both hips and knees and brought them to the chest. - Gluteus: In dorsal decubitus, they flexed only one hip, brought their knee to the chest, and alternated limbs. They took care that the lumbar segment and head remained supported. - Ischiotibial: In dorsal decubitus, the patients flexed their hips and knees, with their feet resting on the mattress. They extended one knee and alternated their limbs. *Session 5* • Activities performed - Repetition of the previous three exercises - Pectoralis: In dorsal decubitus, the patients flexed their hips and knees, with their feet resting on the mattress. They then positioned their arms at approximately 45° abduction and kept the shoulders away from the ears, with the medial epicondyles resting on the mattress and hands open. - Latissimus dorsi: In dorsal decubitus, they flexed their hips and knees, with feet supported on the mattress and lumbar segment in physiological lordosis. They flexed their arms to the maximum while keeping the elbows extended and palms opened. *Session 6* • Activities performed - Repetition of the previous five exercises - Hip adductor: In dorsal decubitus, the patients flexed their hips and knees, joined the soles of their feet, and abducted their thighs. They ensured that the lumbar segment remained in physiological and relaxed lordosis. *Sessions 7–10* Activities performed: All previous exercises

The first three sessions were common to both treatments. The first session was solely educational. The second and third sessions contained body/postural awareness and proprioception exercises, which were preparations for the stretching exercises.

#### Data analysis

Data were analyzed using descriptive and inferential statistics, with *α* < 0.05. Excel for Windows (Microsoft) and SigmaPlot 12.3 (Systat Software Inc.) were used. The variables were tested for normal distributions using the Shapiro–Wilk test.

The differences between groups at posttreatment were analyzed using *t*, chi-square, or Mann–Whitney tests. The differences between the baseline and posttreatment values for each group were analyzed using the paired *t*-test or the Wilcoxon Sign Rank test.

An evaluation of clinically important differences was conducted by estimating the rate of change in the scores of the variable [44]: *Clinical improvement (%) = [(outcome variable score after treatment − outcome variable score at baseline) ÷ (outcome variable score at baseline)].* A cutoff of 30% was defined a priori as the minimal relevant clinical improvement for pain [45]. For the Fibromyalgia Impact Questionnaire score, a 14% improvement was considered relevant for the total score [46]. Meanwhile, in the perception and body self-care reports transcription analysis, we used the domains of the Survey of Pain Attitudes—Brief Version [34] as classes of meanings to describe the perceptions and body self-care reports of the FM patients [47].

## Results

**Fig. 1:**
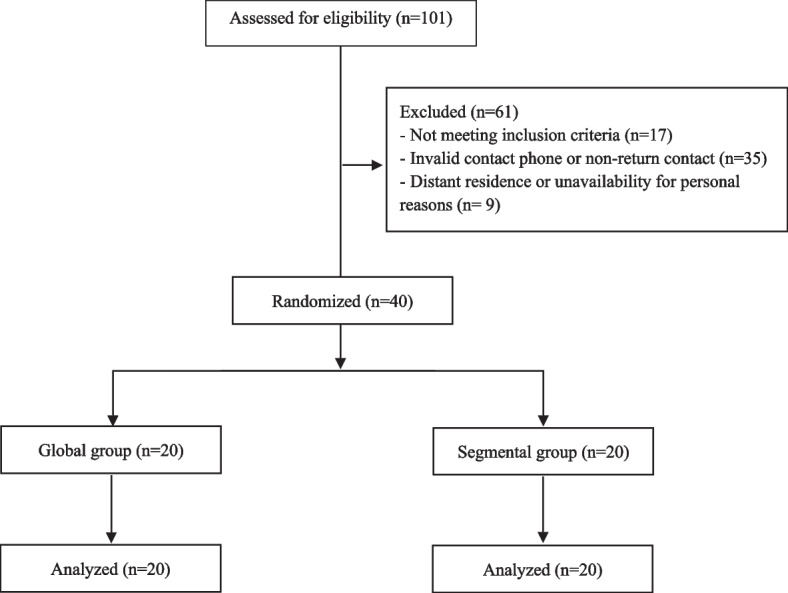
Flow chart for inclusion procedure and allocation of subjects into groups

Of the 101 eligible subjects, 61 were excluded for the reasons described in Fig. 1. Forty participants were randomly assigned to the global and segmental groups, and the data of 40 participants were analyzed. There was only one male participant in the segmental group.

Table 2 shows the demographic and clinical characteristics of the participants under the baseline conditions.

**Table 2:** Demographic and clinical characteristics of the participants at baseline

Characteristics	Global group (*n* = 20)	Segmental group (*n* = 20)
Age (years) (mean ± SD)	50 ± 11	44 ± 13
Education (*n*)		
Primary	4 (20%)	5 (25%)
Secondary	6 (30%)	7 (35%)
University	10 (50%)	8 (40%)
Marital status (*n*)		
Married	13 (65%)	9 (45%)
Not married	7 (35%)	11 (55%)
Occupation (*n*)		
Have a job outdoors	8 (40%)	14 (70%)
Housewife or insurance security	12 (60%)	6 (30%)
BMI (kg/cm^2^)	28.4 ± 5.1	28.2 ± 4.9
Pain duration (years) (median (IQR))	10 (5, 16)	7 (4, 10)
Use medication for pain (n)	16 (80%)	13 (65%)
Pain modulation (*n*)^a^		
Relaxation or rest	14 (70%)	13 (65%)
Medication or physical exercise	9 (45%)	15 (75%)
None	3 (15%)	1 (5%)

*BMI*, body mass index; *SD*, standard deviation; *IQR*, interquartile range

^a^Factors that may decrease pain; subjects might report more than one factor

Table 3 shows the results of the evaluations of the ACR 2011 criteria for FM, pain intensity, pain threshold at tender points, and impact of FM on quality of life at posttreatment. There were no statistically significant differences between the groups.

**Table 3:** Scores for the ACR 2011 criteria, pain intensity, pain threshold, and impact of FM

	Global group (*n* = 20)				Segmental group (*n* = 20)			
	Baseline	Post-treatment	*p*	Clinical improvement	Baseline	Post-treatment	*p*	Clinical improvement
	Mean ± SD/median (IQR)			%	Mean ± SD/median (IQR)			%
ACR 2011 criteria								
WPI	13.0 ± 3.1	4.1 ± 6.0	<0.01	**69**	12.4 ± 3.4	3.0 ± 4.2	<0.01	**76**
SSS	7.9 ± 2.4	2.9 ± 2.9	<0.01	**63**	8.0 ± 2.5	3.3 ± 2.5	<0.01	**59**
VAS Pain (cm)	6.0 ± 1.8	2.2 ± 1.6	<0.01	**63**	6.3 ± 2.1	2.5 ± 1.7	<0.01	**60**
Pain threshold (kg/cm^2^)	1.3 ± 0.8	1.7 ± 0.5	<0.01	31	1.3 ± 0.9	1.9 ± 0.4	<0.01	46
FIQ								
Physical functioning	8.4 ± 7.4	2.1 ± 5.4	<0.01	75	7.4 ± 6.3	1.8 ± 3.9	<0.01	76
Well-being (days)	0 (0, 2)	6 (5, 7) ^a^	<0.01	-	2 (0, 5)	2 (2, 4) ^a^	0.70	-
Work missed (days)	0 (0, 4)	0 (0, 0)	<0.01	-	0 (0, 0)	0 (0, 0)	0.25	-
Job ability (cm)	6.7 ± 2.7	4.0 ± 2.2	<0.01	40	5.8 ± 2.7	3.9 ± 2.1	<0.01	33
Pain (cm)	7.9 ± 1.9	4.0 ± 2.2	<0.01	49	7.1 ± 2.1	4.6 ± 2.0	<0.01	35
Fatigue (cm)	7.0 ± 2.7	3.6 ± 2.5	<0.01	49	8.0 ± 2.1	5.1 ± 2.3	<0.01	36
Morning tiredness (cm)	7.4 ± 2.5	4.1 ± 2.5	<0.01	45	6.9 ± 2.5	5.4 ± 2.5	<0.01	22
Stiffness (cm)	6.3 ± 3.3	3.6 ± 2.2	<0.01	43	6.9 ± 2.6	4.6 ± 2.3	<0.01	33
Anxiety (cm)	6.0 ± 3.2	5.1 ± 2.2	0.11	15	6.1 ± 3.1	5.6 ± 2.8	0.28	8
Depression (cm)	5.7 ± 3.2	4.3 ± 2.4	0.03	25	6.3 ± 2.6	3.3 ± 1.8	<0.01	48
Total score	61.0 ± 19.8	38.2 ± 16.6	<0.01	**37**	57.2 ± 14.0	39.3 ± 11.5	<0.01	**31**

^a^Statistically significant difference between groups at posttreatment (*p* < 0.05)

In bold: relevant clinical improvement

*ACR*, American College of Rheumatology; *WPI*, widespread pain index; *SSS*, symptom severity score; *VAS*, Visual Analog Scale; *FIQ*, Fibromyalgia Impact Questionnaire; *SD*, standard deviation; *IQR*, interquartile range

Table 4 shows the scores for the sensory, affective, and evaluative dimensions of the McGill Pain Questionnaire and the attitudes of patients toward chronic pain from the Survey of Pain Attitudes—brief version at posttreatment. The only statistically significant difference was in the Harm domain of the Survey of Pain Attitudes—brief version, in which the segmental group presented a lower mean value than the global group posttreatment (*p* < 0.05).

**Table 4:** Multidimensionality of pain and attitudes of patients toward chronic pain at baseline and posttreatment

	Global group (*n* = 20)				Segmental group (*n* = 20)			
	Baseline	Post-treatment	*p*	Clinical improvement	Baseline	Post-treatment	*p*	Clinical improvement
	Mean ± SD			%	Mean ± SD			%
McGill Pain Questionnaire								
Sensory	21.2 ± 9.0	14.1 ± 6.9	<0.01	**34**	21.5 ± 5.8	13.1 ± 5.9	<0.01	**39**
Affective	8.0 ± 3.7	4.1 ± 2.1	<0.01	**49**	7.0 ± 2.2	3.0 ± 2.0	<0.01	**57**
Evaluative	3.1 ± 1.6	1.2 ± 1.4	<0.01	**61**	2.9 ± 1.5	1.5 ± 1.5	0.01	**48**
SOPA								
Control	2.4 ± 0.9	3.3 ± 0.6	<0.01	-	2.2 ± 1.4	3.2 ± 0.9	<0.01	-
Emotion	3.3 ± 0.9	3.8 ± 0.4	<0.01	-	3.3 ± 1.0	3.6 ± 0.7	<0.05	-
Disability	3.0 ± 1.2	1.8 ± 1.1	<0.01	-	2.3 ± 1.9	2.3 ± 0.9	0.49	-
Harm	1.4 ± 0.8	1.0 ± 0.6^a^	<0.05	-	1.4 ± 0.8	0.6 ± 0.5 ^a^	<0.01	-
Medication	2.3 ± 1.1	2.0 ± 0.9	0.20	-	2.0 ± 1.0	2.4 ± 1.0	0.26	-
Solicitude	1.4 ± 1.3	1.2 ± 1.2	0.20	-	1.5 ± 1.3	1.0 ± 1.1	0.05	-
Medical cure	2.5 ± 0.9	2.4 ± 0.8	0.32	-	2.7 ± 0.5	2.9 ± 0.8	0.22	-

^a^Statistically significant difference between groups at posttreatment (*p* < 0.05)

In bold: relevant clinical improvement

*SOPA*, Survey of Pain Attitudes-Brief Version

Table 5 shows the body posture measurements, the mCTSIB mean values, and the flexibility data at post-treatment. The only statistically significant difference between groups was for flexibility at the end of treatment (*p* < 0.01), in which the segmental group presented a higher mean value of flexibility at the end of the treatment than the global group.

**Table 5:** Body posture, postural control, and flexibility at baseline and posttreatment

	Global group (*n* = 20)				Segmental group (*n* = 20)			
	Baseline	Post-treatment	*p*	Clinical improvement	Baseline	Post-treatment	*p*	Clinical improvement
	Mean ± SD			%	Mean ± SD			%
Body posture (°)								
Lateral view								
HAH	59.6 ± 15.8	78.1 ± 8.3	<0.01	-	55.6 ± 10.7	62.3 ± 10.4	<0.01	-
VAT	−2.6 ± 4.4	−5.0 ± 2.1	<0.01	-	−2.4 ± 3.3	−4.0 ± 1.7	<0.05	-
VAB	1.7 ± 2.0	1.2 ± 1.2	0.13	-	1.3 ± 2.0	0.6 ± 0.9	0.07	-
Anterior view								
HAH	−0.6 ± 2.7	−0.1 ± 1.6	0.21	-	0.9 ± 2.4	0.4 ± 1.4	0.12	-
HAA	−0.8 ± 2.4	0.0 ± 1.3	<0.05	-	−0.2 ± 1.2	−0.8 ± 0.9	<0.05	-
HAASIS	1.1 ± 1.4	0.2 ± 0.8	<0.05	-	1.1 ± 2.1	0.8 ± 0.8	0.30	-
Postural control (°/s)								
mCTSIB mean	0.70 ± 0.29	0.51 ± 0.08	<0.01	27	0.60 ± 0.12	0.49 ± 0.06	<0.01	18
Flexibility (cm)	16.3 ± 8.6	14.9 ± 4.4^a^	0.23	-9	18.1 ± 10.4	20.4 ± 5.5 ^a^	0.12	13

^a^Statistically significant difference between groups at posttreatment (*p*<0.01)

*HAH*, horizontal alignment of head; *VAH*, vertical alignment of head; *VAT*, vertical alignment of trunk; *VAB*, vertical alignment of body; *HAA*, horizontal alignment of acromion; *HAASIS*, horizontal alignment of anterior superior iliac spine; *mCTSIB mean*, The Modified Clinical Test of Sensory Interaction on Balance Mean

Attitudes and beliefs toward chronic pain improved in both groups at the end of treatment in relation to the following domains: control, emotion, and physical harm (Table 4). In the analysis of the transcription of the reports, we used these domains as classes of meanings to describe the patients’ perceptions and body self-care reports at the end of treatment (Table 6).

**Table 6:** Patients’ perceptions and body self-care reports at the end of treatments

**Classes of meanings**	**Global group**	**Segmental group**
Control: Personal influence on pain management		
	“I take care of my body as best I can; and I take care of my posture in the same way.”	“I know how to control pain better now, I know how to avoid things now that I didn’t avoid before (...) Now when I go to bend over, I do it more carefully. That way, I can avoid hurting so much. I’m much more careful.”
Emotion: Emotions influence the painful experience		
	“Depression, you lying on the bed, hurts your back, so it really needs to be battled.”	“I feel more comfortable with the exercises. It’s really comfortable, that’s the word. Then I think that for greater comfort I forget a little about the pain (...). I feel more at the center. I’m enjoying it. I’m having fun. I feel more excited.
Physical harm: Chronic pain is not hurting itself, and exercise should not be avoided		
	“I’ve already walked this week, which I couldn’t do [before]. Go out for a walk on the street.”	“My body is lighter. It was very rigid [before]. I am also getting to know myself better. In need of maintenance, I’ve learned to do the exercises.”

## Discussion

Both the global and segmental groups presented a decrease in pain intensity, as evaluated by the visual analog scale and the McGill Pain Questionnaire; a decrease in the impact of FM on quality of life; an improvement in the pain threshold at tender points; and improvements in patients’ attitudes toward chronic pain and postural control at the end of treatment. There were no significant differences between the groups.

These were two treatment protocols created specifically for individuals with FM. To our knowledge, this is the first randomized trial to investigate the global posture reeducation method for adults with FM. The duration of the interventions was 10 weeks; each session was approximately 60 to 80 min long, in accordance with a previous FM study [48]. The frequency of sessions was once a week, given that transportation within the large metropolitan area of São Paulo, Brazil, may be difficult for some participants.

Loduca and Samuellan [49] stated that when individuals feel pain, their bodies give signs of the presence of this discomfort by adopting postures. In the current study, it was observed that pain and posture could be modified by stretching exercises. The initial hypothesis was that only the global group would have a change in body alignment. However, both groups showed improvement in head and trunk alignment, particularly in terms of their body axis, without significant differences between them. Thus far, there is still no consensus in the literature on the relationship between improvements in posture and decreases in pain. A systematic review by Sheikhhoseini et al. [50] provides moderate to strong evidence that therapeutic exercises may improve posture. The review’s results indicate that no randomized clinical trial has established a mediation between the change in forward head posture as a cause of pain improvement. In this case, other factors related to the exercises could reduce the pain, not necessarily postural change.

Segmental exercises are static muscle stretching exercises that involve stretching a single muscle or small group of muscles up to a tolerable point and sustaining the position for a certain period, usually 30 s [51]. The other method is based on global posture reeducation, which consists of active progressive stretching of the muscular chains for over 15 min. In our study, both treatments followed the same principles: stretching the same group of muscles, avoiding compensatory motions, maintaining a slow breathing pace, and respecting patients’ limits. Individual sessions allowed for individualized attention from the same therapist, improving the establishment of links to reach therapy goals, as in Maluf et al. [52].

FM patients present deficits in postural control, sensory organization, and balance self-efficacy associated with pain [41]. According to Maluf and Marques [53], orthostatic body posture requires the functional integration of synergistic antigravity muscle chains. By intervening in the muscle chains, a consequent adaptation may have occurred and modified postural control. The reduction in pain in both groups could also explain the improvement in postural control at the end of the treatment.

In addition, the segmental group presented a higher mean value of flexibility than the global group at the end of the treatment. Myofascial tensions may have been distributed through the muscle chains [53], resulting in a decrease in flexibility in the global group at the end of the treatment.

The most recent evidence- and consensus-based FM management guidelines recommend beginning with patient education and physical therapy [54]. García-Ríos et al. [55] defined “patient education” as “any set of educational activities planned by qualified professionals and aimed at improving a patient’s health behaviors and/or health status, and has a specific objective to inform and restructure the perceptions regarding the disorder. This therapy approach is based on the premise that a better understanding of the nature of their disorder may result in improved patient outcomes.” The central focus of patient education is to assume that the patient is an active information processing agent and not just a passive reagent. This point of focus can explain why the results of our study were favorable.

In Brazil, a very recent multidisciplinary educational health promotion program called “Fibro Friends” was validated for individuals with FM [56]. A recent systematic review verified the effectiveness of interdisciplinary education programs for FM [57]. The topics that were most frequently considered in the interdisciplinary health education programs were general information about FM, body practices, physical activities, and pharmacological approaches. The authors concluded that an interdisciplinary health education program decreases pain and improves the quality of life for FM patients.

Part of the educational approach is the ability to care for and be attentive to and welcoming of the patient. This certainly influenced the results of the current study. For example, the following adverse event was considered and prevented: “A possibility of slight discomfort during exercises, which is expected at the start of treatment and should decrease in a few days.” This adverse event was not observed. A welcoming reception is one of the most relevant suggestions included in the National Humanization Policy of the Brazilian National Health System. A welcoming reception expresses an action of approximation or “being with,” that is, an attitude of inclusion. This refers to a commitment to recognizing the other person, such as by showing an attitude of welcoming the patient in his or her pains, joys, ways of living, and feelings [58]. In addition, it brings to the relationships the invention of strategies that can contribute to the dignity associated with their lives and ways of living. Considering the pain and distress related to FM, a welcoming reception of the patient was a priority in the treatment.

In addition, learning positive feelings and understanding promote resilience [59] and reduce stress [60]. Part of the process of building resilience is gaining a sense of hope and purpose [61]. Thus, it has a direct influence on health behaviors and depression.

In chronic pain, the presence of dysfunctional beliefs and negative thoughts are common. Beliefs are culturally shared intimate conceptions or convictions about our perceptions of ourselves, others, and our environment [49]. In the current study, FM patients’ attitudes and beliefs toward chronic pain improved in both groups, and they became more adapted to chronic pain at the end of treatment in relation to the following domains: “Control” refers to how much the patients believe they can control their pain, “emotion” refers to how much the patients believe that their emotions influence their painful experiences, and “physical harm” refers to how much the patients believe that pain means they are hurting themselves and that they should avoid exercise [34]. These changes may have occurred due to the strategies of the educational approach used in this study.

We decided to include the ACR 2011 criteria indexes as outcome measures because these criteria are indicated for clinical and epidemiological research [30, 62]. In our study, the blind evaluator administered the questionnaire to the patients so that their levels of education did not lead to biased results. For both groups, the clinical improvement was approximately 70% on the WPI and 60% on the SSS. To the best of our knowledge, there have been no studies about minimally relevant clinical improvements for the WPI and SSS. We considered a provisional benchmark for interpreting changes in the WPI and SSS of ≥50% as “substantial improvement.” This benchmark was based on a consensus statement on the clinical importance of treatment outcomes in chronic pain clinical trials [45].

The two stretching exercise methods and learning processes could have modulated the generation of pain via the concept of nociplastic pain in FM [22]. The pain threshold at tender points did not increase to the level considered normal. Thus, we observed a situation in which there was still hypersensitivity to palpation due to the presence of tender points (according to the 1990 classification criteria) but few places of spontaneous pain (according to the 2010/2011 and 2016 diagnostic criteria). This means that if we considered the 1990 classification criteria, patients would still have FM, but according to the latest diagnostic criteria of 2010/2011 and 2016, they would no longer have FM. These data should be carefully considered.

### Limitations of the study

Global posture reeducation is a muscle stretching exercise method that is essentially passive and static compared with other exercises, such as aerobic and strengthening exercises.

## Conclusions

Muscle stretching exercises based on global posture reeducation and segmental muscle stretching exercises, both used in concert with an educational approach rooted in cognitive behavioral therapy, reduced the pain intensity and the impact of FM on quality of life and improved the FM patients’ pain threshold at tender points, attitudes toward chronic pain, and postural control. The two methods presented similar effects.

## Data Availability

The datasets generated and/or analyzed in the current study are available from the corresponding author on reasonable request.
